# Gα_i2_- and Gα_i3_-Specific Regulation of Voltage-Dependent L-Type Calcium Channels in Cardiomyocytes

**DOI:** 10.1371/journal.pone.0024979

**Published:** 2011-09-26

**Authors:** Sara Dizayee, Sonja Kaestner, Fabian Kuck, Peter Hein, Christoph Klein, Roland P. Piekorz, Janos Meszaros, Jan Matthes, Bernd Nürnberg, Stefan Herzig

**Affiliations:** 1 Department of Pharmacology, University of Cologne, Cologne, Germany; 2 Department of Biochemistry and Molecular Biology II, University Hospital and Clinics, University of Düsseldorf, Düsseldorf, Germany; 3 Department of Pharmacology and Toxicology, Interfaculty Center of Pharmacogenomics and Pharmaceutical Research, University of Tübingen, Tübingen, Germany; Georgia State University, United States of America

## Abstract

**Background:**

Two pertussis toxin sensitive G_i_ proteins, G_i2_ and G_i3_, are expressed in cardiomyocytes and upregulated in heart failure. It has been proposed that the highly homologous G_i_ isoforms are functionally distinct. To test for isoform-specific functions of G_i_ proteins, we examined their role in the regulation of cardiac L-type voltage-dependent calcium channels (L-VDCC).

**Methods:**

Ventricular tissues and isolated myocytes were obtained from mice with targeted deletion of either Gα_i2_ (Gα_i2_^−/−^) or Gα_i3_ (Gα_i3_^−/−^). mRNA levels of Gα_i/o_ isoforms and L-VDCC subunits were quantified by real-time PCR. Gα_i_ and Ca_v_α_1_ protein levels as well as protein kinase B/Akt and extracellular signal-regulated kinases 1/2 (ERK1/2) phosphorylation levels were assessed by immunoblot analysis. L-VDCC function was assessed by whole-cell and single-channel current recordings.

**Results:**

In cardiac tissue from Gα_i2_^−/−^ mice, Gα_i3_ mRNA and protein expression was upregulated to 187±21% and 567±59%, respectively. In Gα_i3_^−/−^ mouse hearts, Gα_i2_ mRNA (127±5%) and protein (131±10%) levels were slightly enhanced. Interestingly, L-VDCC current density in cardiomyocytes from Gα_i2_^−/−^ mice was lowered (−7.9±0.6 pA/pF, n = 11, p<0.05) compared to wild-type cells (−10.7±0.5 pA/pF, n = 22), whereas it was increased in myocytes from Gα_i3_^−/−^ mice (−14.3±0.8 pA/pF, n = 14, p<0.05). Steady-state inactivation was shifted to negative potentials, and recovery kinetics slowed in the absence of Gα_i2_ (but not of Gα_i3_) and following treatment with pertussis toxin in Gα_i3_^−/−^. The pore forming Ca_v_α_1_ protein level was unchanged in all mouse models analyzed, similar to mRNA levels of Ca_v_α_1_ and Ca_v_β_2_ subunits. Interestingly, at the cellular signalling level, phosphorylation assays revealed abolished carbachol-triggered activation of ERK1/2 in mice lacking Gα_i2_.

**Conclusion:**

Our data provide novel evidence for an isoform-specific modulation of L-VDCC by Gα_i_ proteins. In particular, loss of Gα_i2_ is reflected by alterations in channel kinetics and likely involves an impairment of the ERK1/2 signalling pathway.

## Introduction

G protein-mediated signalling plays a central role in regulation of cardiomyocyte function. Heterotrimeric G proteins consist of three subunits, Gα, Gβ, and Gγ. Agonist-occupied receptors induce dissociation of GDP from and binding of GTP to the G protein α subunit, resulting in G protein activation. Activated Gα and Gβγ subunits couple to a plethora of effectors, including enzymes and ion channels, and hence are involved in many regulatory processes [1], [2]. The role of stimulatory G_s_ and inhibitory G_i_ proteins in cardiac signalling pathways is well studied [3], [4]. Alterations of Gα_i_ protein expression levels are found in heart disease [5], and heart failure in humans leads to upregulation of Gα_i2_ and Gα_i3_ [6], [7], [8], [9]. Whether the upregulation of Gα_i2_ and Gα_i3_ in cardiomyocytes is causative, adaptive, or maladaptive still remains unclear.

Cardiac calcium channels are key components in complex signal transduction pathways and play an essential role in cardiac excitability and in coupling excitation to contraction [10]. One major pathway regulating calcium channels is mediated *via* G protein-mediated signalling. In the heart, the main sarcolemmal calcium channel is the voltage-dependent L-type calcium channel (L-VDCC). This channel is composed of three different subunits. The α_1_ subunit represents the pore forming subunit which contains the voltage sensor and the binding sites for calcium channel modulators [11]. It associates with two auxiliary subunits, β, and α_2_δ [12]. The functional properties of the pore forming subunit are differentially modified due to interaction with various β subunit isoforms [13], [14], [15], [16]. Furthermore, receptor activated Gα_s_ protein stimulates L-VDCCs via adenylyl cyclase-mediated increases in cAMP levels and protein kinase A (PKA) activity [3]. Activation of G_i_ or G_o_ modifies channel function *via* diverse signal cascades [17]. Thus, G protein signalling pathways are crucial in determining and balancing cardiomyocyte function *in vivo*.

In a previous study we addressed the role of Gα_i2_ in β_2_-adrenergic receptor-mediated signalling. Gene deletion of Gα_i2_ in mice reduced single L-VDCC activity in β_2_-adrenergic receptor-transgenic mice [18], whereas pertussis toxin (PTX) treatment reversed this effect. We speculated that this unexpected effect of PTX may have been caused by inhibiting an upregulated Gα_i3_. Recently, Zuberi et al. [19] showed that Gα_i2_ knockout leads to increased L-VDCC mRNA expression and a propensity towards ventricular arrhythmia. Muscarinic receptor-mediated inhibition of L-VDCC activity has been reported to depend on Gα_i2_ but not Gα_i3_ [20]. Though strongly suggested by these data, subtype-specific effects on cardiac L-VDCC by the highly homologous Gα_i2_ and Gα_i3_ isoforms remain unclear so far. Therefore, the present work was undertaken to elucidate whether the effects of these Gα_i_ proteins are redundant or distinct. Using cardiomyocytes from mice lacking Gα_i2_ or Gα_i3_ and wild-type (WT) control animals, we determined structural and functional changes. Further, we examined specific signalling pathways implicated in cardiac L-VDCC modulation by Gα_i_ protein. In this work, we provide evidence that the L-VDCC activity and kinetics are regulated in a non-redundant manner and we support this idea by demonstrating subtype-specific activation of the extracellular signal-regulated kinases 1/2 (ERK1/2) signalling cascade.

## Results

### Gα_i2_ deficiency decreases, while Gα_i3_ deficiency increases L-VDCC current density

**Figure 1:**
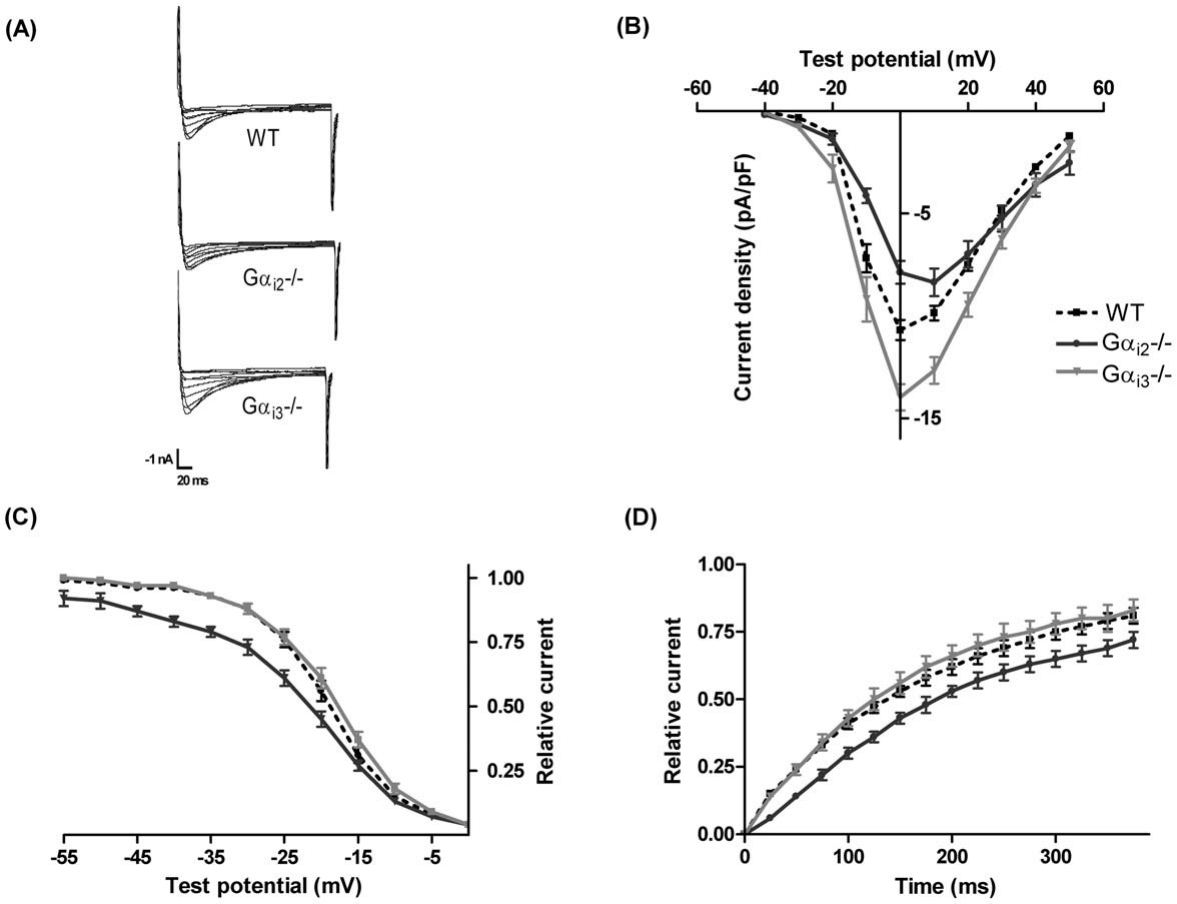
Cardiac whole-cell L-type calcium currents. Representative original current traces obtained at different test potentials (A) and IV-curves (B) reveal an increase of calcium current in ventricular myocytes from mice lacking Gα_i3_ and a decrease in mice lacking Gα_i2_ as compared to WT mice. (C) Steady-state inactivation of Gα_i2_^−/−^ (n = 11) is shifted to more negative voltages as compared to Gα_i3_^−/−^ (n = 13, p<0.05) and WT (n = 18, p<0.05). (D) A slowing of the recovery time constant τ is found in cardiomyocytes from Gα_i2_^−/−^ mice (287±21 ms, n = 9) as compared to Gα_i3_^−/−^ cells (n = 9) and WT (n = 16).

To assess consequences of selective deletion of Gα_i2_ or Gα_i3_ genes, we measured whole-cell L-VDCC currents in cardiomyocytes from WT, Gα_i2_^−/−^, and Gα_i3_^−/−^ mice. Currents recorded at different test potentials are shown as representative original recordings and current-voltage diagrams of summarized data in Fig. 1A and B, respectively. In cardiomyocytes of Gα_i2_^−/−^ mice, the calcium current density at 0 mV was slightly but significantly reduced (−7.9±0.6 pA/pF, n = 11, p<0.05) compared to WT (−10.7±0.5 pA/pF, n = 22). In contrast, current density in cardiomyocytes from Gα_i3_^−/−^ mice was increased (to −14.3±0.8 pA/pF, n = 14, p<0.05 vs. WT). Of note, the peak current in Gα_i2_^−/−^ cardiomyocytes is shifted towards higher voltages. Comparison of time-dependent inactivation by fitting revealed no alterations of fast and slow time constants in all genotypes (e.g. at 0 mV, τ_fast_: WT = 18.4±1.0 ms, Gα_i2_^−/−^ = 26.9±4.0 ms and Gα_i3_^−/−^ = 15.1±2.1 ms; τ_slow_: WT = 95.3±4.2 ms, Gα_i2_^−/−^ = 97.5±17 ms and Gα_i3_^−/−^ = 94.9±9.9 ms; n = 10–13). To test whether changes in gating account for the observed differences in current density, we examined kinetic and steady state properties of activation and inactivation.

### L-VDCC kinetics are altered by Gα_i2_ deletion, but not by Gα_i3_ deletion

The steady-state inactivation properties (Fig. 1C) in Gα_i2_^−/−^ cardiomyocytes were altered compared to WT cells as reflected by a significant leftward shift of V_0.5_ (Gα_i2_^−/−^: −23.4±1.0 mV, n = 11, WT: −19.2±0.7 mV, n = 18) and an increased slope factor (Table 1). In addition, recovery from inactivation was slowed in cardiomyocytes from mice lacking Gα_i2_ (τ: 287±21 ms, n = 9, p<0.05) in comparison to WT animals (τ: 215±14 ms, n = 16; Fig. 1D). In contrast, whole-cell currents in cells form Gα_i3_^−/−^ mice were indistinguishable from WT regarding both steady state inactivation (V_0.5_: −18.2±0.9 mV, n = 13, Table 1) and recovery from inactivation (τ: 203±24 ms, n = 9). Thus, currents from Gα_i2_^−/−^ mice show altered kinetic properties and this might explain the decreased current density described above (Fig. 1). Despite increased current density no alteration of current kinetics in Gα_i3_^−/−^ myocytes was visible. Thus we analyzed single-channel activity to elucidate the opposing effects seen on whole-cell currents in Gα_i2_^−/−^ and Gα_i3_^−/−^ cardiomyocytes.

**Table 1:** Steady-state inactivation parameters.

Parameter	WT	Gα_i2_^−/−^	Gα_i3_^−/−^
V_0.5_ (mV)	−19.1±0.7	−23.4±1.0* †	−18.2±0.9
Slope factor (mV)	5.2±0.2	8.7±0.6* †	5.4±0.2

In Gα_i2_^−/−^ cardiomyocytes V_0.5_ of the steady-state inactivation is significantly shifted to more negative potentials. In addition, Boltzmann fits revealed a significant flattening of the curves as indicated by increased slope factors.

*p<0.05 vs. WT,

† p<0.05 vs. Gα_i3_^−/−^.

### No major changes in gating properties of single L-VDCC in Gα_i3_^−/−^ cardiomyocytes

**Figure 2:**
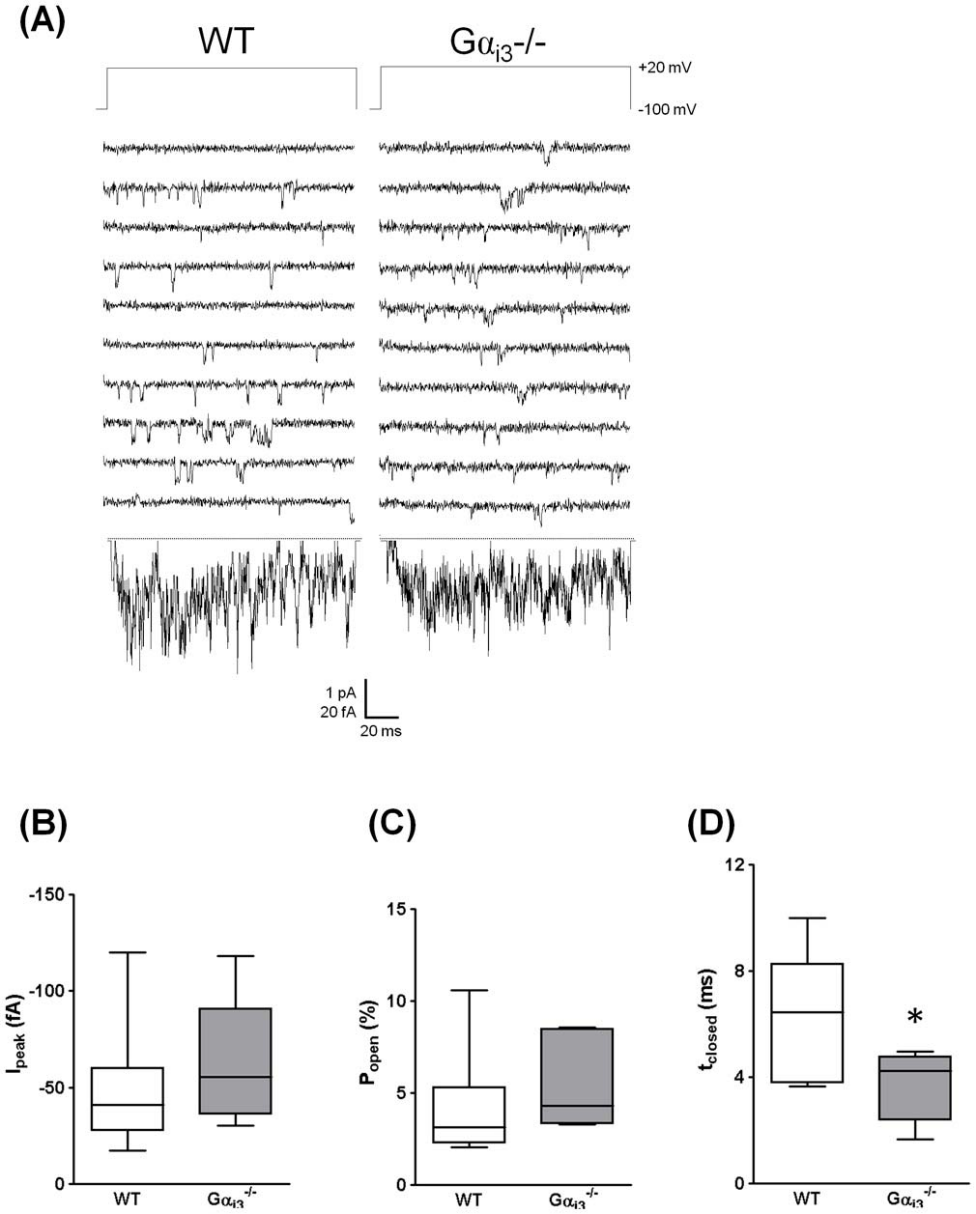
Single-channel properties of L-type calcium channels. (A) Exemplary traces of barium currents show increased single-channel activity in ventricular myocytes of Gα_i3_^−/−^ animals vs. WT mice. (B) The peak ensemble average current is −61±13 fA in Gα_i3_^−/−^ (n = 6) and −44±9 fA in WT mice (n = 7). (C) The open probability within active sweeps is slightly enhanced in Gα_i3_^−/−^ (5.4±1.0% vs. 4.0±0.9% WT) whereas (D) the mean closed time is significantly reduced (Gα_i3_^−/−^ 3.8±0.5 ms vs. 6.6±0.9 ms WT). Unitary amplitude was not different with −0.83±0.02 pA (WT) and −0.79±0.03 pA (Gα_i3_^−/−^). *p<0.05 vs. WT. Box-and-whisker plots indicate minimum and maximum values as well as 25^th^, 50^th^ and 75^th^ percentiles.

Single-channel current recordings in Gα_i3_^−/−^ cardiomyocytes revealed a trend towards increased peak ensemble average currents (Fig. 2B) and higher open probability (Fig. 2C) when compared to WT cardiomyocytes (Table 2). These effects are based on a significant reduction of the mean closed time (3.8±0.5 ms vs. 6.6±0.9 ms, n = 6–7, p<0.05; Fig. 2D). Together with a significantly decreased slow time constant of the closed state and by trend a reduced first latency (Table 2) our single-channel data suggest that in Gα_i3_^−/−^ exit from deeper closed states of L-VDCC is facilitated. Given that single-channel activity in Gα_i2_^−/−^ mice was decreased by trend [18] our findings presented here suggest that Gα_i2_^−/−^ or Gα_i3_^−/−^ knockout leads to distinct changes of cardiac L-VDCC properties. Yet, these only slight functional changes alone do not elucidate the more remarkable augmentation of calcium current density in Gα_i3_^−/−^. Since the remaining Gα_i_ isoform might have compensated for effects of Gα_i3_ deficiency we next checked the expression levels of Gα_i2_ in Gα_i3_ knockouts and *vice versa*.

**Table 2:** Single-channel gating in WT and Gα_i3_^−/−^ myocytes.

Parameter	WT	Gα_i3_^−/−^
**i [pA]**	−0.81±0.02	−0.80±0.03
**I_peak_ [fA]**	−48±13	−61±12
**f_active_ (%)**	76±4	83±7
**P_open_ (%)**	4.2±1.1	5.4±1.0
**t_open_ [ms]**	0.48±0.06	0.29±0.02*
**τ_open_ [ms]**	0.42±0.05	0.26±0.03*
**t_closed_ [ms]**	6.5±0.9	3.8±0.5*
**τ_closed,1_ [ms]**	0.34±0.02	0.36±0.04
**τ_closed,2_ [ms]**	15.5±1.5	10.2±1.7*
**proportion (τ_closed,1_∶τ_closed,2_)**	2.0±0.4	2.2±0.3
**fl [ms]**	27.1±4.1	18.6±2.4

Analysis of single-channel gating parameters in ventricular myocytes from WT (n = 7) and Gα_i3_^−/−^ (n = 6) mice. Recordings with more than one channel were excluded from the analysis. i: unitary current; I_peak_: peak ensemble average current; f_active_: fraction of traces showing at least one opening; P_open_: open probability in active traces; t_open_: mean duration of openings; τ_open_: dwell time constant of the open state; t_closed_: mean duration between two consecutive openings; τ_closed,1_: dwell time constant of the fast closed time component; τ_closed,2_: dwell time constant of the slow closed time component; proportion: ratio of events conferring to either the fast or the slow closed time component; fl: mean latency until the first opening.

*: p<0.05 vs. WT.

### Enhanced expression levels of remaining Gα_i_ isoform

**Figure 3:**
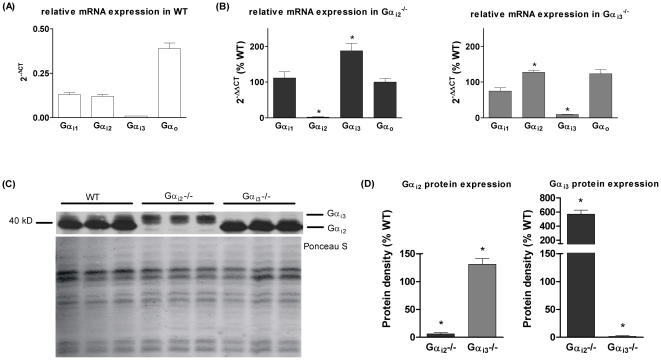
RNA expression levels of cardiac Gα_i/o_ isoforms measured by real-time PCR. GAPDH was used as endogenous control, and WT mice as calibrator (expression = 100%; n = 4). (A) 2^−ΔCT^ values were calculated to analyze the relative expression of Gα_i/o_ isoforms in WT cardiomyocytes. (B) The relative mean expression (2^−ΔΔCT^) reveals a significantly increased Gα_i3_ mRNA content in ventricular tissue of Gα_i2_^−/−^ mice (n = 3) and significantly increased Gα_i2_ mRNA levels in Gα_i3_^−/−^ mice (n = 4). (C) Representative example of Gα_i2_ and Gα_i3_ protein expression in murine ventricular tissue from WT, Gα_i2_^−/−^ and Gα_i3_^−/−^ mice. Cell membranes were isolated and Gα_i_ proteins were analyzed by immunoblotting using an anti-Gα_common_ antibody. Shorter exposure times were used to analyse Gα_i2_ protein. (D) Summarized protein expression data show an upregulation of Gα_i3_ in Gα_i2_^−/−^ mice and an upregulation of Gα_i2_ in Gα_i3_^−/−^ mice (n = 8). *p<0.05 vs. WT.

If the generally accepted assumption holds true that Gα_i2_ and Gα_i3_ proteins are functionally redundant in the heart, we could expect a compensatory upregulation of the remaining Gα_i_ subunit after knockout of the other. We first determined mRNA expression levels for Gα_i1_, Gα_i2_, Gα_i3,_ and Gα_o_ in samples from WT, Gα_i2_^−/−^, and Gα_i3_^−/−^ mice using real-time PCR. In ventricular tissue from WT mice, transcripts for all Gα_i_ protein isoforms and Gα_o_ were found in different amounts (Fig. 3A). While Gα_i2_ and Gα_i3_ are known to be expressed in cardiomyocytes - with Gα_i3_ mRNA being clearly less abundant -, Gα_i1_ and Gα_o_ are likely transcribed in non-cardiomyocyte ventricular cells [21]. Deletion of Gα_i2_ enhanced the mRNA level of Gα_i3_ (to 187±21%, n = 3, p<0.05 vs. WT), as expected [6], [22]. In cardiac tissue of Gα_i3_^−/−^ mice Gα_i2_ mRNA levels were upregulated to only 127±5% (n = 4, p<0.05 vs. WT). No significant changes in Gα_o_ or Gα_i1_ mRNA expression levels were detected, which is in line with the assumption, that these G proteins are not expressed in cardiomyocytes (see above and Fig. 3B).

Next, protein expression of Gα_i_ isoforms was analyzed in cell membrane preparations from WT, Gα_i2_^−/−^, and Gα_i3_^−/−^ ventricles by probing with Gα_common_ antibodies subsequent to high resolution urea/SDS-PAGE-separation of proteins. WT mice expressed both Gα_i2_ and Gα_i3_ subunits with the protein level of Gα_i2_ being much higher than that of Gα_i3_ (Fig. 3C), in line with previous studies (e.g. [22]). As expected, in hearts from Gα_i2_^−/−^ mice Gα_i3_ was found and Gα_i2_ was absent while in hearts from Gα_i3_^−/−^ mice, only Gα_i2_ was detectable (Fig. 3C). The specificity of the detected Gα_i2_ and Gα_i3_ protein bands in Gα_i2_^−/−^, Gα_i3_^−/−^ and WT cardiac tissue was confirmed by analyzing the expression of Gα_i_ isoforms by PTX-mediated [^32^P]ADP ribosylation [23] (data not shown). These experiments prove that gene deletion indeed led to loss of the Gα subunit. Statistical analysis of relative expression levels obtained from immunoblots (Fig. 3D) show that Gα_i2_ protein is significantly upregulated in ventricles obtained from Gα_i3_^−/−^ mice (to 131±10%, n = 8, p<0.05 vs. WT). However, Gα_i3_ protein is much more markedly upregulated upon Gα_i2_ deficiency (to 567±59%, n = 8, p<0.05 vs. WT). Taken together, knockout models examined in this study feature a protein upregulation of the respective other Gα_i_ isoform. These data, together with quantitative mRNA data reported above, suggest that increased levels of Gα_i2_ or Gα_i3_ may partially compensate for the loss of the deleted other Gα_i_ isoform. Hence, any effect seen so far could be caused by the loss of one Gα_i_ protein and/or compensatory signalling exerted by the other. Unfortunately, double-deficient (Gα_i2_^−/−^/Gα_i3_^−/−^) mice are not viable [22] and hence cannot be used to directly address this question. However, all G_i_ proteins can acutely be inactivated by PTX treatment.

### Acute G_i_ inactivation induces L-VDCC kinetic alterations in Gα_i3_^−/−^ cardiomyocytes

**Figure 4:**
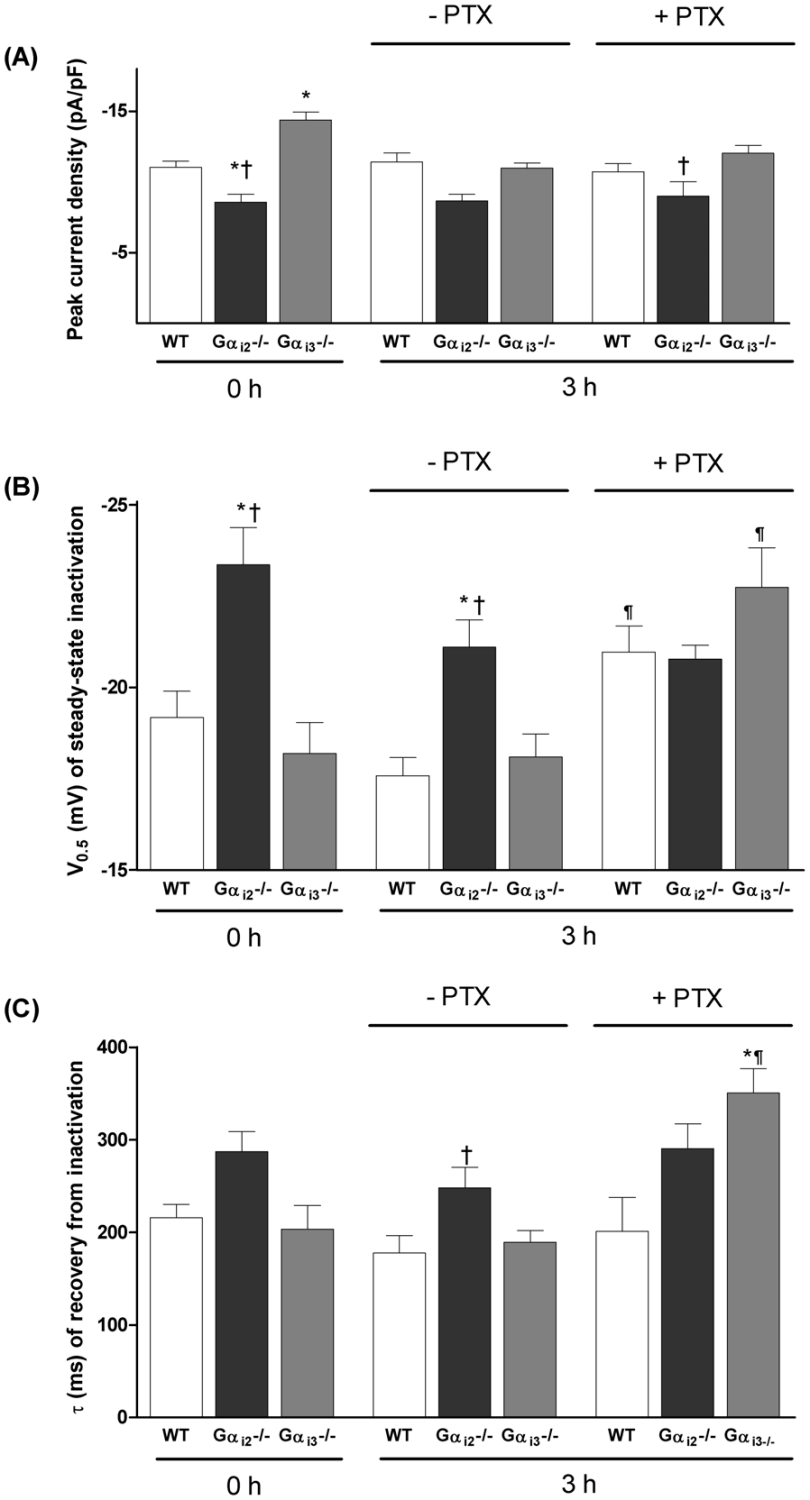
Effects of acute inactivation of G_i/o_ proteins by PTX incubation of isolated cardiac myocytes. (A) Effects of PTX on peak L-VDCC current density. PTX treatment by itself did not affect calcium current density. (B) Effects of PTX on steady-state inactivation, as gauged by the midpoint voltage V_0.5_ of a Boltzmann function. No change is seen after 3 hours of drug-free incubation compared to 0 hour. PTX leads to a significant leftward shift of V_0.5_ in WT (from −19.2±0.7 mV to −21.0±0.7 mV, n = 7–18) and Gα_i3_^−/−^ (from −18.2±0.7 mV to −22.7±1.1 mV, n = 11–13). (C) PTX affects the recovery of the L-VDCC from inactivation. PTX inhibits the channel recovery in Gα_i3_^−/−^ (τ from 189±12 ms to 350±26 ms, n = 5–11). *p<0.05 vs. WT, ^†^p<0.05 vs. Gα_i3_^−/−^, ^¶^p<0.05 vs. 3 h without PTX.

We performed experiments with PTX for acute inactivation of G_i/o_ proteins in cardiomyocytes of WT and Gα_i_-deficient mice. Freshly isolated cardiomyocytes were incubated at 37°C with or without PTX for 3 hours and afterwards maintained and examined at room temperature. It has been shown that this protocol completely ablates G_i_-mediated signalling, e.g. when triggered by adenosine receptors [24]. Of note, the 3 hours incubation phase – even in the absence of PTX – reduced current density in Gα_i3_-deficient cells (Fig. 4A). Notably, similar to WT cells, PTX treatment in Gα_i3_^−/−^ myocytes induced a shift of V_0.5_ for steady state inactivation to more negative potentials (to −22.7±1.1 mV, n = 11; Fig. 4B). Likewise, the recovery from inactivation in Gα_i3_^−/−^ cardiomyocytes was slowed by PTX treatment (to τ: 350±27 ms, n = 8 p<0.05 vs. WT; Fig. 4C). In contrast, the calcium current phenotype in Gα_i2_^−/−^ cells was preserved with time and resistant to treatment with PTX. To summarize, PTX alters channel regulation in cells from Gα_i3_^−/−^ but not from Gα_i2_^−/−^ mice. In Gα_i3_^−/−^, acute PTX treatment – and thus inactivation of Gα_i2_^−/−^ – mimics the kinetic changes induced by chronic gene deletion of Gα_i2_^−/−^. In contrast, the effect of Gα_i3_ gene deletion appears to be transient and does not interfere with channel kinetics. Because some of the calcium current changes reported here may be caused by (long-term) structural alterations rather than (acute) functional modulation of calcium channels, we next examined channel composition.

### No significant structural modification of L-VDCC

**Figure 5:**
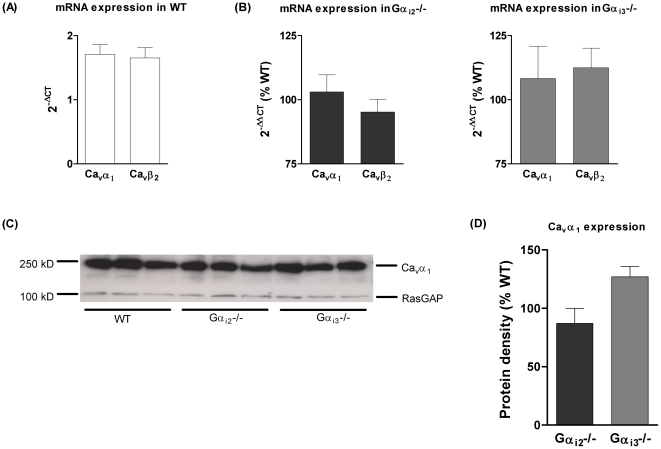
Calcium channel subunit expression. (A) 2^−ΔCT^ values were calculated against GAPDH expression to analyze the relation of L-type calcium channel subunits in WT cardiomyocytes (n = 4). (B) No significant changes in the relative mean expression (2^−ΔΔCT^) were observed in ventricular tissues from Gα_i2_^−/−^ (n = 3) and Gα_i3_^−/−^ mice (n = 4). (C) Representative example of Ca_v_α_1_ protein expression in murine ventricular tissue from WT, Gα_i2_^−/−^ and Gα_i3_^−/−^ mice. (D) Quantification of Ca_v_α_1_ protein levels show no change in ventricles from Gα_i3_^−/−^, Gα_i2_^−/−^ and WT mice (n = 3).

To obtain insight into the effects of specific and constitutive Gα_i_-deficiency on L-VDCC structure and expression, we determined RNA expression levels of the pore forming Ca_v_α_1_ subunit and the predominant murine cardiac Ca_v_β subunit, Ca_v_β_2_ [14], [16], [25], which is involved in calcium channel trafficking and gating [26], [27], [28]. The Ca_v_β_2_ subunit mRNA expression in ventricles from Gα_i2_^−/−^ and Gα_i3_^−/−^ animals is not altered compared to WT expression profile (Fig. 5B). In Gα_i2_^−/−^ and Gα_i3_^−/−^ ventricles, the pore forming Ca_v_α_1_ subunit mRNA (Fig. 5B) and membrane protein (Fig. 5C and D) expression levels exhibit only slight and insignificant changes. Thus, these data do not suffice to explain the Gα_i_ isoform-specific regulation of the current density. Therefore, we next switched to the posttranslational level and addressed signalling pathways that are involved in G_i_ protein-mediated action.

### Akt activation is unaltered by deletion of Gα_i2_ or Gα_i3_

**Figure 6:**
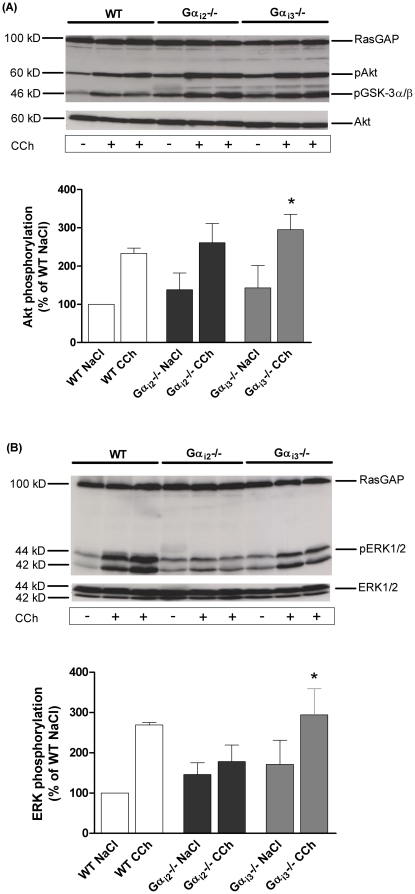
Signalling events downstream of Gα_i_ stimulation. Representative western blot of cardiac tissues of animals treated either with saline or CCh (0.5 mg/kg body weight) for 15 min. (A) Western Blot and densitometry analyses of ventricular homogenates show that Akt phosphorylation was significantly and to a similar extent increased after CCh stimulation in all genotypes (n = 3). Total amount of Akt protein was unaltered and was used as loading control. (B) Western blot showing ERK1/2 phosphorylation and total ERK1/2 expression and bar graphs of combined results expressed as increase in pERK1/2 normalized to total ERK1/2 and compared to saline treated WT mice. The increase of ERK1/2 activation in CCh treated cells was markedly inhibited by Gα_i2_ gene deletion compared to WT and Gα_i3_^−/−^ mice (n = 3). *p<0.05 vs. WT NaCl.

Cardiac Gα_i_ is known to activate the PI3-kinase Akt/PKB pathway [29], and Akt-mediated β-subunit phosphorylation prevents Ca_v_α_1_ degradation [30]. A G_i_-isoform-specific regulation of Akt could explain the calcium current increase in the case of Gα_i3_^−/−^ and decrease in case of Gα_i2_^−/−^. To examine the functional significance of Gα_i_-dependent activation of Akt *in vivo*, animals were treated with either saline or the muscarinic receptor agonist carbachol (CCh, 0.5 mg/kg body weight) by i.p. injection; 15 min later animals were killed, and signalling activity was assessed in heart preparations. Phosphorylation of Akt and its downstream effector glycogen synthase kinase-3α/β (GSK3α/β), were increased in cardiac tissue from all mouse strains after treatment with CCh (Fig. 6A). Although the basal phosphorylation of Akt in Gα_i2_^−/−^ and Gα_i3_^−/−^ cardiac tissue was slightly increased compared to WT (to 138±44% and 143±58% of WT, respectively), we could not observe statistically significant differences between CCh stimulated WT (233±14% to basal), Gα_i2_^−/−^ (201±27%) and Gα_i3_^−/−^ (195±25%) mice (each n = 3). Importantly, the total amount of Akt was not changed in all mice models. Thus, G_i_-subtype specific channel regulation seems to be independent of Akt phosphorylation in the investigated knockout models.

### Lack of Gα_i2_ protein abolishes ERK1/2 activation

Recently, a marked increase of L-type calcium channel density that involved PKC-dependent activation of the ERK1/2 pathway was reported [31]. To determine its involvement, we measured the activation of ERK1/2 protein in total cardiac tissue. Gα_i2_^−/−^ mice demonstrated a significantly blunted increase of ERK1/2 phosphorylation in CCh-stimulated animals (128±5% to basal, n = 3) compared to WT (268±6%, n = 3) and Gα_i3_^−/−^ (192±20%, n = 3) cardiomyocytes. Of note, in Gα_i2_^−/−^ and Gα_i3_^−/−^ cardiac tissues, the basal ERK1/2 phosphorylation levels were increased compared to WT basal phosphorylation (Fig. 6B), while the total amount of ERK1/2 in the heart was the same in all mouse models tested. These results indicate a Gα_i2_-dependent ERK1/2 phosphorylation and strongly suggest that ERK1/2 plays an important role in isoform-specific Gα_i_ protein signalling.

## Discussion

The two inhibitory G protein isoforms G_i2_ and G_i3_ are both upregulated in heart failure [6], [7], [8]. One functionally important target of G_i_ protein signalling is the L-VDCC, the crucial trigger of cardiac excitation-contraction coupling. G_i_-protein-mediated inhibition of L-VDCC has been demonstrated for β_2_-adrenergic [4] and muscarinic [20] receptor signalling. In this context, we previously provided single-channel evidence that Gα_i2_ does not confer the L-VDCC inhibition observed in mice with chronic overexpression of the β_2_-adrenergic receptor [18]. On the other hand, cardiac Gα_i2_ (but not Gα_i3_) seems necessary and sufficient to mediate the muscarinic receptor-mediated L-VDCC inhibition [20], presumably through the classical adenylyl cyclase pathway. So far, no isoform-specific function could be assigned to cardiac Gα_i3_; however, G_i3_ has been shown to be an exclusive and specific regulator of autophagy in the liver [22], [32].

The different behaviour of L-VDCC currents obtained with isolated myocytes from Gα_i2_^−/−^ and Gα_i3_^−/−^ mice shown here and previously [18] demonstrates contrasting functional roles of these two Gα_i_ isoforms. Although the issue has also been addressed by others [19], [20], [33], our study is the first to demonstrate small but significant changes of basal whole-cell current density: a reduction in myocytes from Gα_i2_^−/−^ mice and an increase in myocytes from Gα_i3_^−/−^ mice. The altered steady-state inactivation and recovery observed with Gα_i2_^−/−^ under basal conditions (Fig. 1C and D), and with Gα_i3_^−/−^ myocytes after PTX treatment (Fig. 4B and C), point to a modulation of gating properties specific to Gα_i2_. In contrast to data presented here, Nagata *et al.* [20] did not detect a significant difference in L-VDCC activity between myocytes from WT, Gα_i2_^−/−^ and Gα_i3_^−/−^ mice. With respect to Gα_i2_^−/−^, this finding can be explained by the different prepulse potentials: the prepulse voltages (−50 mV used by Nagata and co-workers vs. −40 mV in our case) - intended to inactivate primarily sodium currents - lie within the descending part of steady-state inactivation (Fig. 1C). This likely translates into the more reduced peak in the current voltage plot in our study. Interestingly, Zuberi *et al.* [19] compared Gα_i2_^−/−^ mice with Gα_i1_^−/−^/Gα_i3_^−/−^ double knockout mice and found distinct effects on surface ECGs: in Gα_i2_^−/−^ animals (but not in the double knockout mice), the effective refractory period was reduced and ventricular arrhythmias were induced more easily. In summary, Gα_i2_ protein deletion showed dramatic consequences on channel regulation *in vivo* and *ex vivo*.

There are no changes of cardiac L-VDCC composition regarding the main cardiac L-VDCC subunits, Ca_v_α_1_ and Ca_v_β_2_ (Fig. 5B and D) that would explain the obtained effects on current density. Furthermore, because of the compensatory upregulation of the remaining Gα_i_ isoform in case of either Gα_i2_ or Gα_i3_ deficiency (Fig. 3B and D), it is difficult to attribute the observed changes in L-VDCC regulation/function to the higher expression of one Gα_i_ isoform or to the loss of the other or to both. Therefore, given the novel functional effects reported here for cardiac Gα_i2_ and Gα_i3_, we have to consider a number of molecular pathways. For instance, activation of Stim1 [34] may lead to altered L-VDCC function and subcellular distribution. Enhanced endocytosis and degradation of calcium channels can also be mediated by activation of PIKfyve [35] and subsequent Ca_v_α_1_ targeting to lysosomes. Further, the RGK proteins Rad and Rem expressed in the heart [36] are appealing candidates, because they negatively regulate both membrane expression and gating of L-VDCC [37], [38] while little is known about how these small GTP-binding proteins are regulated themselves [39]. In our present study, we focused on two other important molecular pathways since they are significantly regulated by G_i_-signalling. First, PI3-kinase Akt/PKB signalling is known to be activated by cardiac Gα_i_ [29], and Catalucci *et al.* [30] revealed a mechanism through which the PI3-kinase Akt/PKB pathway modulates Ca^2+^ entry in cardiac cells via L-VDCC. Our data showed a CCh-induced Akt phosphorylation independent of the deletion of either Gα_i_ isoform (Fig. 6A). Second, it was demonstrated that deactivation of G_i_ leads to a significant reduction in ERK1/2 phosphorylation and that this effect was G_s_- and G_q_- independent [29], [40]. In the present study, we have shown that Gα_i2_ deletion prevents phosphorylation of ERK1/2. Recently, Smani *et al.* [31] found a leftward shift and a marked increase in L-VDCC density induced by urocortin, which involved PKC-dependent activation of the MAPK-ERK1/2 pathway. Based on these findings we propose that loss of activation of L-VDCC by ERK1/2 might be a mechanism involved in functional regulation of calcium current in Gα_i2_ deficient mice.

A change of Gα_s_ mediated signalling might account for altered calcium currents when a Gα_i_ is lacking. With this caveat in mind, we demonstrated in our previous study that Gα_s_ protein expression remained unaffected in hearts from mice deficient in the major isoform Gα_i2_ [18]. Yet, possible changes in associated proteins like Gβγ subunits might indirectly affect Gα mediated signalling [41]. Indeed, we observed that expression of Gβ_1/2_ was slightly reduced in Gα_i2_^−/−^, but not Gα_i3_^−/−^ (data not shown). In any case, our single-channel analysis (in particular, the decrease in open time) does not support the idea of enhanced Gα_s_ mediated, cAMP-mediated signalling in Gα_i3_-deficient hearts (Table 2).

The data presented here could not elucidate all effects seen in the knockout animals. Thus, eventually, G_i3_'s role in L-VDCC regulation remains unclear, mainly in light of the absence of acute PTX effects in Gα_i2_^−/−^ mice. However, due to the effects seen by incubation without PTX, our findings suggest that the increment in channel activity observed in the absence of Gα_i3_ might be driven by an *in vivo* mechanism, which is not preserved *ex vivo* (Fig. 4A). It has also to be pointed out that the immunoblot data reveal total cardiac membrane channel protein levels, which does not necessarily match up the fraction of functional channels located in the sacrolemma. Given our currently available methods to analyze the subcellular localization of calcium channels and their regulation, all of these ideas require further work, which for technical reasons has to be done in recombinant systems.

Taken together, our data reported here and in a previous paper [18] point to the (patho-) physiological importance of subtype-specific G_i_ protein signalling in the heart. In particular, in terminal heart failure, Gα_i2_ upregulation now appears as an attractive mechanism linked to remodelling of L-VDCC [13], [14], [15], [16]. Therefore, the present study provides new insights into potential mechanisms linking modulation of L-VDCC to the inhibitory G protein isoform G_i2_ in cardiomyocytes, and highlights G_i2_-specific signalling via ERK1/2. Further research needs to focus on detailed signalling pathways involving ERK1/2.

## Methods

### Ethic statement

Animal breeding, maintenance and experiments were approved by the responsible federal state authority (Landesamt für Natur-, Umwelt- und Verbraucherschutz Nordrhein-Westfalen; reference No. K 27, 24/04 and 8.87-51.05.20.09.232) and the responsible local authority (Umwelt- und Verbraucherschutzamt der Stadt Köln; reference No. 576/1.36.6.3.-47/08 Be). All animal experiments conform with the *Guide for the Care and Use of Laboratory Animals* published by the US National Institutes of Health (NIH Publication No. 85-23, revised 1996).

### Animals

Generation, breeding and characterization of Gα_i2_- or Gα_i3_- deficient mice have been described previously [18], [22], [42], [43], [44], [45]. All Gα_i_-deficient mouse strains used were backcrossed onto a C57Bl6 background for >10 generations. Knockout and WT control mice were maintained at the animal facilities of the Heinrich-Heine-University, Düsseldorf, and of the Department of Pharmacology at the University of Cologne. Mice analyzed in this study were of both sexes, 3–9 months of age and weighted 20–35 g.

### Genotyping

Tail-clip analysis was performed on 3–4 weeks old mice. Genomic DNA was prepared and genotyping PCR for Gα_i2_ and Gα_i3_ was performed as described previously [18], [43].

### Real time PCR

Primer for Gα_i1_, Gα_i2_, Gα_i3_ isoforms and Ca_v_α_1_ subunit were described previously [25], [46]. Specific primers for the Gα_o_ and Ca_v_β_2_ subunit were designed using Primer Express Software v3.0 (Applied Biosystems, Foster City, USA). For Gα_o_: 5′-TGGCATCGTAGAAACCCACTT-3′ (sense) and 5′-CGACGTCAAACAGCCTGAAG-3′ (antisense) and for Ca_v_β_2_: 5′-GGGAGGCAGTACGTAGAGAAGCT-3′ (sense) and 5′-TGCAAATGCAACAGGTTTT GTC-3′ (antisense). Total cellular RNA was extracted from murine heart (ventricle) according to the manufacturer's protocol (Qiagen QIAshredder and RNeasy Mini Kit, Qiagen, Hilden, Germany). For qualitative analysis of RNA integrity, 2 µg of total RNA was separated on a 1% formaldehyde agarose gel. Total RNA was subsequently converted into cDNA by ImProm-II Reverse Transcription Kit (Promega, Mannheim, Germany). Real-time PCR was carried out using the 7500 Real-Time PCR system (Applied Biosystems) under standard conditions with 200 pM PCR primers. Each sample was analyzed in triplets using SYBR green (Applied Biosystems) as fluorescent detector and GAPDH as endogenous control.

### Cell membrane preparation

Murine cardiac ventricles were disrupted and homogenised in a lysis buffer containing 10 mM Tris-HCl (pH 7.4), 1 mM EDTA, 0.5 mM DTT, and an EDTA-free protease inhibitor cocktail (Roche, Penzberg, Germany) using an ultra-turrax blender. Cellular membranes were isolated by two steps of centrifugation at 450 g and 30.000 g. Membrane pellets were subsequently dissolved in a buffer consisting of a freezing supplement (70 mM Tris (pH 7.4), 12 mM MgCl_2_, 60% Glycerol, 240 µg/µl DNAse) and the lysis buffer in a ratio of 1∶6 in order to stabilize membrane-associated proteins.

### Phosphorylation assay

Animals were injected i.p. with 0.5 mg/kg carbachol (CCh) diluted in 0.9% normal saline. Sham injections were performed with 400 µl 0.9% saline per 30 g weight. After 15 min ventricular tissue was harvested and lysed in buffer (50 mM HEPES, 1% Triton, 50 mM NaCl, 10 mM Na_3_VO_4_, 0.1% SDS, 0.1 M NaF, 10 mM EDTA, complete mini protease inhibitor, pH 7.4) and left for 30 min at 4°C. Total cell lysates were extracted from the supernatant by centrifugation at 13.500 g for 20 min.

### Immunoblotting

Gα_i_ and Ca_v_α_1_ proteins isolated from cell membranes were separated on 6 M urea/9% SDS-PAGE gels (protein content per lane was 90 µg) and on 8% SDS-PAGE gels (protein content per lane was 100 µg), respectively [22]. Protein kinase B/Akt and ERK1/2 in total cell lysates were separated on 10% SDS-PAGE gels (100 µg protein content per lane). Separated proteins were blotted onto nitrocellulose membranes (Hybond C extra; Amersham Bioscience). Gα_i_ proteins were detected with an anti-Gα_common_ antibody [45] (1∶1000) and Ca_v_α_1_ with an anti-Ca_v_α_1_ antibody (1∶200; Sigma Aldrich). For detection of phosphorylation, membranes were incubated with phospho-Akt (Ser473), phospho-GSK3α/β (Ser21/9) and phospho-ERK1/2 (Thr202/Tyr204) antibodies. Membranes were reprobed with Akt and ERK1/2 antibodies (each 1∶1000; Cell Signalling) after stripping. Emitted light of stained membranes were captured on films and developed in different expositions. Protein densities were calculated using Aida Image Analyzer (Raytest, Straubenhardt, Germany) software. The Ras-GTP-activating protein (RasGAP; [47]) was used as a loading control for Ca_v_α_1_. Equal loading on blotting membrane for Gα_i_ proteins was controlled by a non-specific protein staining using Ponceau S. Only blots with equal loading were analyzed. To confirm Gα_i_ band specificity, we performed ADP ribosylation of PTX-sensitive G proteins as described [23].

### Cardiomyocyte isolation

Single ventricular myocytes were isolated from hearts of 3–9 months old mice by enzymatic dissociation using a method described previously [48]. Only rod shaped cardiomyocytes were used for the experiments. Cells were maintained at room temperature and subjected to patch-clamp analysis. If indicated, a fraction of isolated ventricular myocytes was incubated without or with 1.5 µg/ml of PTX (Sigma Aldrich, St. Louis, USA) for 3 hours at 37°C [24].

### Single-channel measurements

Single-channel patch clamp recordings were done in the cell-attached configuration as reported [18]. The composition of bath solution was (mM): K-glutamate 120, KCl 25, MgCl_2_ 2, HEPES 10, EGTA 2, CaCl_2_ 1, Na_2_-ATP 1, glucose 10 (pH 7.4 with KOH). Patch pipettes (7–9 MΩ) contained (mM): BaCl_2_ 70, HEPES 10, sucrose 110 (pH 7.4 with TEA-OH). Barium currents were recorded at room temperature using a holding potential of −100 mV and depolarizing test pulses to +20 mV (duration 150 ms, frequency 1.66 Hz, 180 sweeps per experiment minimum). Data were sampled at 10 kHz and filtered at 2 kHz using an Axopatch 200A amplifier (Axon Instruments, Sunnyvale, CA, U.S.A.). Only experiments with one single active channel in the patch were analyzed (identified by the lack of stacked openings).

### Whole-cell current measurements

Conventional whole-cell patch clamp recordings were performed with cells maintained at room temperature in bath solution containing (mM): NaCl 137, CsCl 5.4, CaCl_2_ 2, MgCl_2_ 1, glucose 10, HEPES 10 (pH 7.4 with NaOH). Pipette (2–3 MΩ) solution was composed of (mM): CsCl 120, MgCl_2_ 1, Mg-ATP 4, EGTA 10, HEPES 5 (pH 7.2 with CsOH). Giga-Ohm seals (resistance 2–5 GΩ) were formed by gentle suction. At the beginning of each experiment, membrane capacitance was measured by means of fast depolarizing ramp pulses from −80 to −85 mV over 25 ms. Cells were depolarized from a holding potential of −80 mV to a 50 ms prepulse to −40 mV in order to inactivate sodium channels. This was followed by test pulse voltages ranging from −40 to +50 mV in 10 mV steps (pulse duration 150 ms). For time-dependent inactivation, the declining raw currents at −10, 0, +10, +20 and +30 mV were fitted by a double-exponential function, yielding fast and slow time constant. For investigation of gating kinetics, standard two-pulse protocols were used: the voltage-dependent inactivation was measured after a prepulse of variable amplitude and 250 ms duration, followed by a test pulse of fixed amplitude for 50 ms. The midpoint voltage V_0.5_ was determined by fitting a Boltzmann function to the data. Recovery from inactivation was determined by two 200 ms depolarizing voltage pulses to 0 mV. The interpulse interval at a holding potential of −45 mV was increased from 50 to 375 ms in 25 ms steps. Recovery was fitted by a mono-exponential function, yielding the recovery time constant τ.

### Statistical analysis

Data are presented as means ± SEM. Patch-clamp data were analyzed using pClamp software (CLAMPEX 6 and FETCHAN, Axon Instruments). Analysis of L-VDCC kinetics was performed as described previously [49] using GraphPad Prism 4. A one-sample t-test was used to analyze normalized data. Differences between genotypes were analyzed by one-way ANOVA followed by Dunnett's or Tukey's post test, and between treatments within one given genotype by two-tailed Student's t tests. For electrophysiological statistics, number of cardiomyocytes from a minimum of 3 animals, and for molecular biology statistics, number of animals was evaluated. P values<0.05 were considered statistically significant.
