# Correction: Gα_i2_- and Gα_i3_-Specific Regulation of Voltage-Dependent L-Type Calcium Channels in Cardiomyocytes

**DOI:** 10.1371/annotation/7be097c0-9075-41ae-91e9-d2209de952cc

**Published:** 2013-08-15

**Authors:** Sara Dizayee, Sonja Kaestner, Fabian Kuck, Peter Hein, Christoph Klein, Roland P. Piekorz, Janos Meszaros, Jan Matthes, Bernd Nürnberg, Stefan Herzig

An author was omitted from the list.

The ninth author should be Lutz Bjrnbaumer.

The affiliation of this author is: Laboratory of Neurobiology, National Institutes of Health, 111 TW Alexander Drive, North Carolina, United States of America

Additional Funding information for this author is: Research supported in part by the Intramural Research Program of the NIH, Project 201-ES-101643.

The Author Contributions of this author were: contributed reagents/materials analysis tools. 

